# Recurrent Leflunomide-Induced Drug Reaction With Eosinophilia and Systemic Symptom (DRESS) Syndrome Despite Prolonged Steroid Taper: A Case Report

**DOI:** 10.7759/cureus.29319

**Published:** 2022-09-19

**Authors:** Scott Gayfield, Joshua Busken, Sarmed Mansur

**Affiliations:** 1 Internal Medicine, The University of Toledo, Toledo, USA

**Keywords:** adverse drug reaction, rheumatoid arthritis, leflunomide, drug-induced hypersensitivity syndrome (dihs), drug reaction with eosinophilia and systemic symptoms (dress)

## Abstract

Drug reaction with eosinophilia and systemic symptoms (DRESS) is a severe adverse drug reaction characterized primarily by nonspecific systemic symptoms such as fever, a classical rash, and eosinophilia. While this is an adverse reaction more often related to medications such as anticonvulsants, many drugs have been reported to be implicated in this event. We report a case of a 35-year-old male who developed DRESS syndrome within one month of beginning leflunomide therapy. Despite treatment with a prolonged steroid taper, he developed a flare-up with transaminitis less than two months after his initial hospitalization. Our patient was managed with steroid pulse therapy and cyclosporine, which resulted in an improvement of symptoms and transaminitis. To our knowledge, only nine previous cases of leflunomide-induced DRESS syndrome have been previously reported.

## Introduction

Drug reaction with eosinophilia and systemic symptoms (DRESS), also known as drug-induced hypersensitivity syndrome (DIHS) is a severe adverse drug reaction that occurs most often within two to six weeks of initiating therapy of the offending agent [1]. While there are various manifestations of DRESS syndrome, the most common presentation is a rash that most often begins as maculopapular eruptions that coalesce with time [2]. Systemic symptoms can also range and most commonly include fever, malaise, and eosinophilia. However, other extracutaneous organs can be involved, including most often the liver [3].

While DRESS syndrome is most often caused by medications such as anticonvulsants and sulfonamides, many other drugs have been reported to induce the reaction [1]. However, few cases have been reported of leflunomide-induced DRESS syndrome. We present a case of a young male with a history of seropositive rheumatoid arthritis (RA) who, after being diagnosed with leflunomide-induced DRESS syndrome approximately four to six weeks prior to admission, developed a recurrent DRESS syndrome with transaminitis despite prolonged steroid therapy.

## Case presentation

The patient is a 35-year-old male (weight 62.4 kg, BMI 21.5 kg/m^2^) with a past medical history significant for seropositive RA diagnosed one year prior to admission who presented to the emergency department with diffuse pruritic rash and joint pain. Notably, the patient’s RA had previously been managed by hydroxychloroquine (HCQ) monotherapy after his initial diagnosis one year prior to admission. Three months prior to admission, the patient was started on combination therapy of HCQ + leflunomide (LEF), which the patient stated provided significant benefit to his joint pain and range of motion. One month following the initiation of HCQ+LEF therapy, the patient presented to an outside hospital with symptoms of fever, myalgia, and a diffuse rash. Following work-up at this outside hospital and in the setting of beginning LEF approximately one month prior to hospitalization, the patient was diagnosed with LEF-induced DRESS syndrome with liver injury. This hospital stay included cholestyramine wash-out therapy, precipitate cholestyramine-facilitated removal of the drug, and high-dose steroid treatment. The patient was discontinued from the LEF due to DRESS syndrome. HCQ was also discontinued due to the liver injury associated with the DRESS syndrome. The patient was discharged home on a prolonged prednisone taper, starting at 60 mg once daily. Official records of laboratory and pathology results were unable to be obtained from this initial hospitalization, so the basis of this diagnosis was unable to be confirmed. Following hospitalization, the patient had a resolution of subjective symptoms and the rash. One month after initial hospitalization, the patient reported that he began to feel flu-like symptoms, including subjective fever and myalgia, and developed a rash four to five days later. During this time, the rash continued to worsen over the course of a month, which resulted in an emergency department visit and subsequent work-up.

Upon presentation, the patient complained of a generalized pruritic rash, diffuse joint tenderness, back pain, and heartburn. The initial physical exam was significant for facial edema and a diffuse erythematous maculopapular rash that affected approximately 50% of his body including the arms, hands (including palms), legs, stomach, back, hands, feet (including soles), face, and neck without any evidence of blisters, wounds, skin sloughing. He was hemodynamically stable upon presentation with a temperature 98.0 °F, blood pressure 142/89, respiratory rate 18 breaths/minute, heart rate 108 beats/minute, and O_2_ saturation 99% on room air. Initial labs were significant for alanine transaminase (ALT) 184 U/L (reference range: 0-40 U/L), aspartate transaminase (AST) 77 U/L (reference range: 0-41 U/L), white blood cells (WBC) 13.03 x 10^9^/L (reference range: 4.0-11.0 x 10^9^/L) with neutrophils 86%, lymphocytes 10.3%, monocytes 1.8%, eosinophils and basophils 0%. All other labs of the comprehensive metabolic panel and complete blood count were within normal limits. Importantly, serology for hepatitis A virus (HAV), hepatitis B virus (HBV), and hepatitis C virus (HCV) as well as antinuclear antibody (ANA) were negative and excluded these as causes for liver injury.

While it was noted that the patient's lack of eosinophilia was not characteristic of DRESS syndrome, the Registry of Severe Cutaneous Adverse Reactions (RegiSCAR) scoring system for the diagnosis of DRESS, shown in Table 1, characterized the diagnosis of DRESS in our patient as being “possible” [3]. With this score in the setting of recently diagnosed LEF-induced DRESS syndrome, we considered this to be the most likely diagnosis. Additionally, the rheumatology team noted that recurrences can occur after the initial resolution of the first episode especially when the steroids are tapered too quickly.

**Table 1:** The Registry of Severe Cutaneous Adverse Reactions (RegiSCAR) scoring system for the diagnosis of DRESS. The overall score is added and characterized as follows; final score < 2: excluded, final score 2 to 3: possible, final score 4-5: probable, final score > 5: definite. Included is a scoring system for our specific patient, which indicated a final score of 2. DRESS: drug reaction with eosinophilia and system symptoms; HAV: hepatitis A virus; HBV: hepatitis B virus; HCV: hepatitis C virus; ANA: antinuclear antibody Adapted from [3].

Clinical Parameters	Score			Patient	Patient Score
	-1	0	+1		
Fever ≥101.3°F (38.5°C)	No/unknown	Yes		No	-1
Lymphadenopathy		No/unknown	Yes	No	0
Eosinophilia ≥0.7 × 10^9^ or ≥10% if leucopenia (Score 2 points of ≥1.5 × 10^9^)		No/unknown	Yes	No	0
Atypical lymphocytes		No/unknown	Yes	No	0
Skin rash suggestive of DRESS (≥2 facial edemas, purpura, infiltration, desquamation)	No	Unknown	Yes	Yes	1
Skin rash involves ≥50% of BSA		No/unknown	Yes	Yes	1
Skin biopsy suggestive of DRESS	No	Yes/unknown		No	-1
Organ involvement(1 point for each organ involved, maximum score of 2)		No	Yes	Yes	1
Disease duration ≥15 days	No/unknown	Yes		Yes	0
Exclusion of other causes (1 points if at least 3 of the following are performed and are negative: HAV, HBV, HCV, mycoplasma, chlamydia, ANA, blood culture)		No/unknown	Yes	Yes	1

The patient was treated inpatient for six days with pulse steroid and cyclosporine therapy. Methylprednisolone 125 mg twice daily was used initially, followed by 500 mg once daily for three days then tapered to 150 mg twice daily for one day prior to discharge. Due to the persistent transaminitis and the recurrence of symptoms despite prolonged corticosteroid therapy, cyclosporine therapy was started at 150 mg twice daily (5 mg/kg/day). As seen in Table 2, the transaminitis improved slightly every day with the patient finally being discharged with an ALT 56 U/L and AST 18 U/L.

**Table 2:** A trend of the patient’s liver function test (LFT) throughout the hospital stay. ALT: alanine transaminase; AST: aspartate transaminase

Day	0	1	2	4	6
ALT	184	166	131	90	56
AST	77	41	26	16	18

The patient was discharged on prednisone 60 mg daily and cyclosporine 150 mg twice daily for one week followed by 75 mg twice daily for another week at which point it would be stopped. The patient was also provided symptom-relief support with diphenhydramine, hydroxyzine, and lanolin topical cream. Outpatient follow-up with the rheumatology clinic for medication reconciliation was scheduled for two weeks following discharge.

## Discussion

DRESS syndrome is a severe adverse drug reaction that occurs in approximately two per 100,000 people per year [4]. However, agents shown previously to have a higher risk of DRESS syndrome, such as anticonvulsants, can have incidences of up to one in 1,000 to one in 10,000 exposures [5]. While the exact mechanism is not entirely understood, DRESS is considered to be a T-cell-mediated hypersensitivity reaction. The two predominating theories are 1) a drug-specific immune response leading to the rapid expansion of activated CD4+ and CD8+ T cells and 2) reactivation of herpesvirus in which the immunodeficient state caused by the offending agent causes viral reactivation and expansion of T cells. The latter of these theories remains controversial, but it should be noted that reactivation of the Herpesviridae family (e.g. HHV-6, HHV-7, Epstein-Barr virus [EBV], and cytomegalovirus [CMV]), most commonly HHV-6, occurs in up to 75% of DRESS patients [6].

LEF is a disease-modifying antirheumatic drug (DMARD) used to treat active RA. While gastrointestinal events such as nausea and diarrhea are the most reported adverse effects of LEF therapy [7], DRESS syndrome is rarely reported. A literature review of PubMed revealed only nine previous cases of LEF-induced DRESS syndrome have been reported [8-12], suggesting that the rate of LEF-induced DRESS syndrome is significantly less than the average incidence.

The clinical presentation of DRESS syndrome varies. The initial prodromal phase is typically characterized by nonspecific symptoms such as fever, chills, myalgias, fatigue, and lymphadenopathy. As the disease progresses, dermatologic manifestations are common. Most often, this presents as a maculopapular rash that begins as one or multiple erosions and progresses to a coalescing rash [2], similar to our patient. Systemic symptoms vary widely, but most often include fever (up to 90%), eosinophilia (up to 95%), leukocytosis (up to 95%), neutrophilia (up to 78%), and lymphadenopathy (up to 65%) [3,13]. Organ injury is another common manifestation of DRESS, most often affecting the liver (53% to 90%) but also the kidney (10% to 30%), lungs (up to 30%), and heart (2% to 20%) [3,14,15]. Additionally, relapses/flare-ups are common after the resolution of initial acute disease, manifesting in up to 25% of patients within weeks to months (median 4.5 months) after initial resolution [16]. Flare-ups occur more often in patients treated with systemic steroids, especially if the steroids are tapered too rapidly [17]. A scoring system for the diagnosis of DRESS, known as RegiSCAR, encompasses these various characteristics and can be used to guide diagnosis [3].

While our patient classically had a subjective fever, maculopapular rash, leukocytosis, neutrophilia, and transaminitis, he did not present with eosinophilia. Although the lack of eosinophilia is uncommon, the RegiSCAR scoring system for DRESS (Table 1) characterized the diagnosis of DRESS in our patient to be “possible” [3]. This score in conjunction with a known history of DRESS syndrome within the past two months made this, we believed, the most likely diagnosis. Notably, the patient had been taking steroids for six weeks by the time he presented, which can prevent eosinophilia. While there is a lack of clinical trial-based evidence on the management of DRESS syndrome, we provided our patient pulse steroid therapy utilizing methylprednisolone. We used the following steroid therapy: methylprednisolone 125 mg twice daily on day one of admission, followed by 500 mg once daily for three days then tapered to 150 mg twice daily for one day prior to discharge on prednisone 60 mg once daily for up to two months, depending on the progression of disease at the follow-up appointment. We also utilized cyclosporine 150 mg twice daily (5 mg/kg/day) therapy for one week followed by 75 mg twice daily for another week, after which it would be stopped. Cyclosporine is typically used for severe organ involvement and patients who did not initially respond to systemic corticosteroids [18], both of which was true in this case. His ALT was 184 U/L on admission, which decreased every day until 56 U/L on discharge (Table 2). Therefore, his ALT measured over two times the upper limit of normal and therefore met criteria for major organ involvement. Additionally, the patient had recurrence of DRESS following a prolonged steroid taper after his last appointment, suggesting that he failed steroid therapy. Intravenous immunoglobulins have also been successfully used [19] but was not utilized in our case. Our patient required a six-day hospital stay which is consistent with the typical hospital stay for DRESS syndrome being 6-17 days (median nine days) [4].

## Conclusions

DRESS syndrome is a T-cell-mediated hypersensitivity characterized by systemic symptoms and classical drug rash. Although it is classically caused by medications such as anticonvulsants, there are several medications that have been associated with this event. To our knowledge, we report the 10th known case of leflunomide-induced DRESS syndrome. While our patient was treated with a combination of steroid pulse and cyclosporine, further research is needed to develop a standardized management plan for DRESS syndrome.

## References

[1] Husain Z, Reddy BY, Schwartz RA (2013). DRESS syndrome: Part I. Clinical perspectives. J Am Acad Dermatol.

[2] Demoly P, Adkinson NF, Brockow K (2014). International consensus on drug allergy. Allergy.

[3] Kardaun SH, Sekula P, Valeyrie-Allanore L (2013). Drug reaction with eosinophilia and systemic symptoms (DRESS): an original multisystem adverse drug reaction. Results from the prospective RegiSCAR study. Br J Dermatol.

[4] Wolfson AR, Zhou L, Li Y, Phadke NA, Chow OA, Blumenthal KG (2019). Drug reaction with eosinophilia and systemic symptoms (DRESS) syndrome identified in the electronic health Record allergy module. J Allergy Clin Immunol Pract.

[5] Knowles SR, Shapiro LE, Shear NH (1999). Anticonvulsant hypersensitivity syndrome: incidence, prevention and management. Drug Saf.

[6] Picard D, Janela B, Descamps V (2010). Drug reaction with eosinophilia and systemic symptoms (DRESS): a multiorgan antiviral T cell response. Sci Transl Med.

[7] Zhu H, Li R, Da Z (2018). Remission assessment of rheumatoid arthritis in daily practice in China: a cross-sectional observational study. Clin Rheumatol.

[8] Mohty R, Chaby G, Pannier M, Defossez C, Denoeux JP, Lok C (2003). Drug hypersensitivity syndrome induced by leflunomide. Ann Dermatol Venereol.

[9] Shastri V, Betkerur J, Kushalappa PA, Savita TG, Parthasarathi G (2006). Severe cutaneous adverse drug reaction to leflunomide: a report of five cases. Indian J Dermatol Venereol Leprol.

[10] Vaish AK, Tripathi AK, Gupta LK, Jain N, Agarwal A, Verma SK (2011). An unusual case of DRESS syndrome due to leflunomide. BMJ Case Rep.

[11] Pinto B, Dhir V, Krishnan S, Nada R (2013). Leflunomide-induced DRESS syndrome with renal involvement and vasculitis. Clin Rheumatol.

[12] Rao S, Sunkara A, Srivastava N, Sampat P, Ramos C, Albert E (2021). An uncommon presentation of DRESS syndrome secondary to leflunomide use: a case report. J Investig Med High Impact Case Rep.

[13] Skowron F, Bensaid B, Balme B (2015). Drug reaction with eosinophilia and systemic symptoms (DRESS): clinicopathological study of 45 cases. J Eur Acad Dermatol Venereol.

[14] Chen YC, Chiu HC, Chu CY (2010). Drug reaction with eosinophilia and systemic symptoms: a retrospective study of 60 cases. Arch Dermatol.

[15] Cacoub P, Musette P, Descamps V, Meyer O, Speirs C, Finzi L, Roujeau JC (2011). The DRESS syndrome: a literature review. Am J Med.

[16] Picard D, Vellar M, Janela B, Roussel A, Joly P, Musette P (2015). Recurrence of drug-induced reactions in DRESS patients. J Eur Acad Dermatol Venereol.

[17] Tetart F, Picard D, Janela B, Joly P, Musette P (2014). Prolonged evolution of drug reaction with eosinophilia and systemic symptoms: clinical, virologic, and biological features. JAMA Dermatol.

[18] Kirchhof MG, Wong A, Dutz JP (2016). Cyclosporine treatment of drug-induced hypersensitivity syndrome. JAMA Dermatol.

[19] Funck-Brentano E, Duong TA, Bouvresse S (2015). Therapeutic management of DRESS: a retrospective study of 38 cases. J Am Acad Dermatol.

